# Genome Sequences of Two Strains of the Food Spoilage Mold Aspergillus fischeri

**DOI:** 10.1128/MRA.01328-19

**Published:** 2019-12-12

**Authors:** Shu Zhao, Jean-Paul Latgé, John G. Gibbons

**Affiliations:** a Department of Food Science, University of Massachusetts, Amherst, Massachusetts, USA; b Molecular and Cellular Biology Graduate Program, University of Massachusetts, Amherst, Massachusetts, USA; c Aspergillus Unit, Institut Pasteur, Paris, France; d Organismic and Evolutionary Biology Graduate Program, University of Massachusetts, Amherst, Massachusetts, USA; Vanderbilt University

## Abstract

Aspergillus fischeri is a common food spoilage fungus and a close relative of the opportunistic human pathogen Aspergillus fumigatus. Here, we sequenced the genomes of two isolates of *A. fischeri* to build resources for comparative genomics and to aid in differentiation between *A. fischeri* subspecies.

## ANNOUNCEMENT

Aspergillus fischeri is a filamentous fungus that is naturally found in soil and other decaying organic matter (1, 2), a common agent of food spoilage (1–3), and a rare opportunistic pathogen in humans (4–6). In contrast, Aspergillus fumigatus is a closely related species but is responsible for hundreds of thousands of infections and deaths each year (7, 8). *A. fischeri* and A. fumigatus diverged as recently as ∼4 million years ago (mya) (9) and share several phenotypic similarities (e.g., thermotolerance, hypoxia tolerance, ascospore formation and morphology, and the production of certain secondary metabolites) (4, 10–12). To date, the genomes of more than 100 A. fumigatus isolates have been fully sequenced (13–17), while only one *A. fischeri* genome has been sequenced (16).

We sequenced the genomes of *A. fischeri* IBT 3003 and IBT 3007, which are part of a collection in the Department of Biotechnology at Technical University of Denmark (Lyngby, Denmark) and were originally isolated from soil in Denmark (10). For each isolate, 1 × 10^6^ spores were inoculated onto potato dextrose agar (PDA) plates at 37°C for 72 hours. DNA was isolated directly from spores using the MasterPure yeast DNA purification kit following the manufacturer’s instructions, with several minor modifications.

The 150-bp paired-end libraries were constructed and sequenced by Novogene on an Illumina NovaSeq 6000 sequencer. Raw sequence reads were first deduplicated using Tally (version 15-065) with “–with-quality” and “–pair-by-offset” options to remove potential PCR duplicates (18). Next, we used Trim Galore (version 0.4.2; http://www.bioinformatics.babraham.ac.uk/projects/trim_galore/) to remove residual adaptor sequences and trim reads at low-quality sites using the parameters “–paired,” “–stringency 1,” “–quality 30,” and “–length 50.” The preassembly-improved data sets consisted of 14,619,651 and 15,944,684 read pairs for *A. fischeri* IBT 3003 and IBT 3007, respectively. These data sets were error corrected and assembled using SPAdes (version 3.9.1) with the “–careful” parameter and k-mer sizes of 31, 43, 73, and 93 (19). The cumulative assembly sizes for *A. fischeri* IBT 3003 and IBT 3007 were 31.61 Mb and 31.25 Mb and the G+C contents were 49.5% and 49.5%, respectively. We extracted the internal transcribed spacer (ITS) region from the *A. fischeri* IBT 3003 and IBT 3007 genomes, and they were subjected to a BLAST search against the NCBI nonredundant database (20). Both ITS sequences shared 100% sequence identity to the reference *A. fischeri* NRRL 181 genome (16). We then used QUAST (version 5.0.2) to assess the quality of the assemblies (21). *A. fischeri* IBT 3003 and IBT 3007 were assembled into 1,037 and 1,193 scaffolds, with *N*_50_ values of 365 kb and 377 kb, respectively. BUSCO (version 3) was used to evaluate the completeness of the genome assemblies, using the “ascomycota_odb9” gene set (22). Totals of 98.4% and 98.5% of BUSCO genes were recovered from the *A. fischeri* IBT 3003 and IBT 3007 assemblies, respectively.

### Data availability.

The whole-genome shotgun projects for *A. fischeri* IBT 3003 and IBT 3007 have been deposited in GenBank under the accession numbers VWYB00000000 and VWYA00000000, respectively. Raw Illumina data have been deposited to the NCBI Sequence Read Archive under the project accession number PRJNA564742.

## References

[1] Pitt JI, Hocking AD 2009 Fungi and food spoilage, vol 519 Springer, New York, NY.

[2] Evelyn KHJ, Silva FVM 2016 Modeling the inactivation of Neosartorya fischeri ascospores in apple juice by high pressure, power ultrasound and thermal processing. Food Control 59:530–537. doi:10.1016/j.foodcont.2015.06.033.

[3] Kavanagh J, Larchet N, Stuart M 1963 Occurrence of a heat-resistant species of Aspergillus in canned strawberries. Nature 198:1322. doi:10.1038/1981322a0.

[4] Mead ME, Knowles SL, Raja HA, Beattie SR, Kowalski CH, Steenwyk JL, Silva LP, Chiaratto J, Ries LNA, Goldman GH, Cramer RA, Oberlies NH, Rokas A 2019 Characterizing the pathogenic, genomic, and chemical traits of Aspergillus fischeri, a close relative of the major human fungal pathogen Aspergillus fumigatus. mSphere 4:e00018-19. doi:10.1128/mSphere.00018-19. 30787113 PMC6382966

[5] Lamoth F 2016 Aspergillus fumigatus-related species in clinical practice. Front Microbiol 7:683. doi:10.3389/fmicb.2016.00683. 27242710 PMC4868848

[6] Balajee SA, Kano R, Baddley JW, Moser SA, Marr KA, Alexander BD, Andes D, Kontoyiannis DP, Perrone G, Peterson S, Brandt ME, Pappas PG, Chiller T 2009 Molecular identification of Aspergillus species collected for the transplant-associated infection surveillance network. J Clin Microbiol 47:3138–3141. doi:10.1128/JCM.01070-09. 19675215 PMC2756904

[7] Brown GD, Denning DW, Gow NA, Levitz SM, Netea MG, White TC 2012 Hidden killers: human fungal infections. Sci Transl Med 4:165rv13. doi:10.1126/scitranslmed.3004404. 23253612

[8] Bongomin F, Gago S, Oladele RO, Denning DW 2017 Global and multi-national prevalence of fungal diseases-estimate precision. J Fungi (Basel) 3:E57. doi:10.3390/jof3040057. 29371573 PMC5753159

[9] Steenwyk JL, Shen XX, Lind AL, Goldman GH, Rokas A 2019 A robust phylogenomic time tree for biotechnologically and medically important fungi in the genera Aspergillus and Penicillium. mBio 10:e00925-19. doi:10.1128/mBio.00925-19. 31289177 PMC6747717

[10] Girardin H, Monod M, Latgé JP 1995 Molecular characterization of the food-borne fungus Neosartorya fischeri (Malloch and Cain). Appl Environ Microbiol 61:1378–1383. 7747958 10.1128/aem.61.4.1378-1383.1995 PMC167394

[11] Dagenais TRT, Keller NP 2009 Pathogenesis of Aspergillus fumigatus in invasive aspergillosis. Clin Microbiol Rev 22:447–465. doi:10.1128/CMR.00055-08. 19597008 PMC2708386

[12] Latgé JP 1999 Aspergillus fumigatus and aspergillosis. Clin Microbiol Rev 12:310–350. doi:10.1128/CMR.12.2.310. 10194462 PMC88920

[13] Nierman WC, Pain A, Anderson MJ, Wortman JR, Kim HS, Arroyo J, Berriman M, Abe K, Archer DB, Bermejo C, Bennett J, Bowyer P, Chen D, Collins M, Coulsen R, Davies R, Dyer PS, Farman M, Fedorova N, Fedorova N, Feldblyum TV, Fischer R, Fosker N, Fraser A, García JL, García MJ, Goble A, Goldman GH, Gomi K, Griffith-Jones S, Gwilliam R, Haas B, Haas H, Harris D, Horiuchi H, Huang J, Humphray S, Jiménez J, Keller N, Khouri H, Kitamoto K, Kobayashi T, Konzack S, Kulkarni R, Kumagai T, Lafon A, Lafton A, Latgé J-P, Li W, Lord A, Lu C, Majoros WH, May GS, Miller BL, Mohamoud Y, Molina M, Monod M, Mouyna I, Mulligan S, Murphy L, O'Neil S, Paulsen I, Peñalva MA, Pertea M, Price C, Pritchard BL, Quail MA, Rabbinowitsch E, Rawlins N, Rajandream M-A, Reichard U, Renauld H, Robson GD, Rodriguez de Córdoba S, Rodríguez-Peña JM, Ronning CM, Rutter S, Salzberg SL, Sanchez M, Sánchez-Ferrero JC, Saunders D, Seeger K, Squares R, Squares S, Takeuchi M, Tekaia F, Turner G, Vazquez de Aldana CR, Weidman J, White O, Woodward J, Yu J-H, Fraser C, Galagan JE, Asai K, Machida M, Hall N, Barrell B, Denning DW 2005 Genomic sequence of the pathogenic and allergenic filamentous fungus Aspergillus fumigatus. Nature 438:1151–1156. doi:10.1038/nature04332. 16372009

[14] Abdolrasouli A, Rhodes J, Beale MA, Hagen F, Rogers TR, Chowdhary A, Meis JF, Armstrong-James D, Fisher MC 2015 Genomic context of azole resistance mutations in Aspergillus fumigatus determined using whole-genome sequencing. mBio 6:e00536-15. doi:10.1128/mBio.00536-15. 26037120 PMC4453006

[15] Takahashi-Nakaguchi A, Muraosa Y, Hagiwara D, Sakai K, Toyotome T, Watanabe A, Kawamoto S, Kamei K, Gonoi T, Takahashi H 2015 Genome sequence comparison of Aspergillus fumigatus strains isolated from patients with pulmonary aspergilloma and chronic necrotizing pulmonary aspergillosis. Med Mycol 53:353–360. doi:10.1093/mmy/myv003. 25851262

[16] Fedorova ND, Khaldi N, Joardar VS, Maiti R, Amedeo P, Anderson MJ, Crabtree J, Silva JC, Badger JH, Albarraq A, Angiuoli S, Bussey H, Bowyer P, Cotty PJ, Dyer PS, Egan A, Galens K, Fraser-Liggett CM, Haas BJ, Inman JM, Kent R, Lemieux S, Malavazi I, Orvis J, Roemer T, Ronning CM, Sundaram JP, Sutton G, Turner G, Venter JC, White OR, Whitty BR, Youngman P, Wolfe KH, Goldman GH, Wortman JR, Jiang B, Denning DW, Nierman WC 2008 Genomic islands in the pathogenic filamentous fungus Aspergillus fumigatus. PLoS Genet 4:e1000046. doi:10.1371/journal.pgen.1000046. 18404212 PMC2289846

[17] Garcia-Rubio R, Monzon S, Alcazar-Fuoli L, Cuesta I, Mellado E 2018 Genome-wide comparative analysis of Aspergillus fumigatus strains: the reference genome as a matter of concern. Genes (Basel) 9:E363. doi:10.3390/genes9070363. 30029559 PMC6071029

[18] Davis MPA, van Dongen S, Abreu-Goodger C, Bartonicek N, Enright AJ 2013 Kraken: a set of tools for quality control and analysis of high-throughput sequence data. Methods 63:41–49. doi:10.1016/j.ymeth.2013.06.027. 23816787 PMC3991327

[19] Bankevich A, Nurk S, Antipov D, Gurevich AA, Dvorkin M, Kulikov AS, Lesin VM, Nikolenko SI, Pham S, Prjibelski AD, Pyshkin AV, Sirotkin AV, Vyahhi N, Tesler G, Alekseyev MA, Pevzner PA 2012 SPAdes: a new genome assembly algorithm and its applications to single-cell sequencing. J Comput Biol 19:455–477. doi:10.1089/cmb.2012.0021. 22506599 PMC3342519

[20] Johnson M, Zaretskaya I, Raytselis Y, Merezhuk Y, McGinnis S, Madden TL 2008 NCBI BLAST: a better Web interface. Nucleic Acids Res 36:W5–W9. doi:10.1093/nar/gkn201. 18440982 PMC2447716

[21] Gurevich A, Saveliev V, Vyahhi N, Tesler G 2013 QUAST: quality assessment tool for genome assemblies. Bioinformatics 29:1072–1075. doi:10.1093/bioinformatics/btt086. 23422339 PMC3624806

[22] Simao FA, Waterhouse RM, Ioannidis P, Kriventseva EV, Zdobnov EM 2015 BUSCO: assessing genome assembly and annotation completeness with single-copy orthologs. Bioinformatics 31:3210–3212. doi:10.1093/bioinformatics/btv351. 26059717

